# Abstracts and Gleanings

**Published:** 1877-11-20

**Authors:** 


### Abstracts and Gleanings.

Antiseptic Surgery.—Lister's system is not the application of peculiar dressings, or the use of paritcular antiseptics. It is a method of treating wounds based on the "germ theory," and its principal aim is to prevent the entrance of germs into wounds, to destroy them if already there, and to guard against the accumulation of wound secretions. In order to attain this end he surrounds the patient with a series of precautionary measures, which have from time to time been improved on by him. These require peculiar materials and appliances, of which I present the latest phase that came under my observation. They are enumerated without particular plan. Where the mode of preparation is not indicated, it will suggest itself; where it is described, it is adapted as much as possible to self-preparation, thereby facilitating the introduction of the system by diminishing its greatest objection—the cost. Their use is described as concisely as possible, and will become clearer when I shall treat of the mode of operating and dressing.

1. Carbolized Solution (Acid, carbol. cryst. 5, aqæ. 100) is used to clean the neighborhood of wounds before operation, to disinfect the hands of surgeon and assistants, and instruments, to wash out septic wounds, and clear drainage tubes.

2. Carbolized Water, a 2½ per cent. solution of crystalized carbolic acid in water. It is used in the spray, and to wet the "lost gauze."

3. Carbolized Oil (Acid. carbol. cryst.

5, ol. oliv. 100), to oil catheters or other instruments, or fingers when about to be introduced into some of the cavities. It is also employed when a constant direct contact of the antiseptic with the wound is necessary, as in caries,—or where the gauze dressing cannot be applied, as in abcess of the rectum.

4. Solution of Chloride of zinc (8 per per cent), 1 part of liq. zinc. chlor. mixed with 3 parts of water. Where wounds have been exposed, unprotected to the access of atmospheric air, or where, from mistake in dressing, aseptic wounds have become septic, they are swabbed out with this solution. It is more effective than carbolic solution, but too powerful for permanent use in the dressings.

5. The Spray. In order to prevent the entrance of living germs, during an operation or dressing, a spray of "carbolized water" is directed on the wound. The best instrument for this purpose is "Lister's spray," a steam atomizer which throws a large cone of finely divided spray. It is almost indispensable in long operations and where a considerable space of tissue is exposed. In its absence it may be replaced by the ordinary steam atomizer, of which two ought to be at hand, as they are sooner exhausted. Their suction tube is unnecessary, and a glass tube, drawn to a fine point, and bent at an acute angle, to throw the steam against wounds without necessity of tipping the instrument, may take its place. In the absence of these, or for short dressings, Richardson's spray apparatus may be used. It has, however, serious defects; it gives out frequently without apparent cause, is very fatiguing, and wets the wounds too much, as the spray is not as finely divided as that of the steam atomizer.

6. The Protective is oiled silk, coated with copal varnish, to render it impermeable, and then covered with a thin layer of 1 part dextrine, 2 parts starch, and 16 parts "carbolized solution," to facilitate adhesion of the disinfecting fluids, into which it is dipped before being applied to the wound. The purpose of the "protective" is to prevent the irritating effect of the contact of the antiseptic with the wound. It is placed immediately over this, overlapping it but little.

7. The Antiseptic Gauze, a peculiar unstarched cotton gauze, selected by Mr. Lister on account of the facility with which secretions penetrate its meshes. It is prepared with antiseptics, and thus, after absorbing the wound fluids, it prevents their decomposition. Pieces of cotton gauze, six yards in length and one yard in width, are to be placed in a zinc trough and heated in the water-bath for several hours, after which they are spread out, and a hot mixture of 1 part cryst. carbolic acid, 5 common resin and 7 paraffine is poured over them by means of a syringe. They are then returned to the trough and submitted to pressure for some hours, to cause an even distribution of the fluids. The resin is to hold the carbolic acid more firmly, and prevent it from being washed out or evaporated too quickly. The paraffine diminishes adhesion of the dressing. This gauze is prepared in factories in Germany, the best known, being the "International" factory, at Schaffhausen, Switzerland.

[The above we clip from *A. C. Girard's report to the Surgeon General, U. S. A.* Other appliances are mentioned as the McIntosh, or common rubber cloth, used to keep the secretions of the wound from finding their way immediately to the surface, and to compel them to permeate the whole dressing, thus being constantly in contact with the carbolic acid.

*The Catgut*, for ligatures and deep sutures; *The Salacylic Cotton; The Sponges; Drainage tubes.* Antiseptic Silk, Boracic Water, 3½ per cent solution of boracic acid in water, used to moisten the *boracic lint.* The *boracic ointment*, used to spread on thin cotton, and applied to ulcers and granulating surfaces.—Ed.]

---

Surgeon-Major Porter's Sawdust Pads.—On May 1st, a male, aged twenty-four, was caught between a fly-wheel in motion and an adjacent wall, so that his left leg was crushed, the bones being fractured in many places, and the soft parts being extensively lacerated. I found it necessary to perform a primary amputation, as low in the thigh as the bruised condition of the soft parts would permit. The stump was drained, and was dressed with carbolized oil on lint, and gutta percha tissue over this. The limb was then swung on a drop-splint, resting on sawdust pads. There was a great deal of blood-stained serum, and afterwards of grumous fluid, which oozed from the bruised tissues of the stump; but the parts of the stump in apposition healed by the first intention. The discharge above mentioned was received on a drop-pad, which was removed daily. The pad which bore the weight of the limb, and the limb itself, were not disturbed for fourteen days. The pad, when removed, was perfectly sweet, and no serum had run into or in any way soiled the bed linen. The patient is now—May 22d, three weeks after the operation—convalescent, having recovered without an unfavorable symptom, only his recovery was slow, owing to the quantity of blood lost whilst being brought up from Blackwall to the hospital. In this instance the pad was most serviceable. It enabled us to leave the thigh undisturbed for the time mentioned; and, from its yielding, it allowed the limb to mould for itself its bearings, which were, throughout, free from all discomfort. With the drop apparatus we were able to dress the stump without causing the patient the slightest pain.

A girl, aged sixteen, required to have her leg amputated immediately above the ankle joint (Syme's operation) on account of carious disease of the left tarsus. The stump was drained, covered with carbolized oil on lint and gutta-percha tissue, and was secured on a drop splint and swung. The small sawdust pad was changed during the first week every second or third day; the larger pad which supported, the leg was not disturbed in any way for three weeks; when removed it was sweet and clean.

Apart from the question under consideration, these cases are of interest with reference to the results obtained in the treatment of severe wounds and extensive suppurations. As to the use of the pads, it may be said that they are approved by the Sisters for their cleanliness, and for the manner in which they keep the bed linen from being soiled by discharge of serum or of pus. They are easily made so as to fit as required, and they are inexpensive. When the quilting is properly attended to, they are comfortable to the patient, readily yielding to such pressure as that, for for instance, caused by the weight of the leg, and moulding so as to give equable support. Whilst they effectually absorb discharge, it is as well, when this is considerable, that the pad should be changed every two or three days, but when, in addition to the pad, carbolized oil dressing is used, they can be left for a longer period. Thus, in the case of the two amputations, the pads which supported the leg in one, and the thigh in the second, were not touched for three weeks, and for fourteen days, respectively. I do not feel disposed to rely entirely upon these pads for keeping parts absolutely clean; but, in conjunction with carbolized oil, or with some kindred dressing, they are amongst the best pads with which I am acquainted, and I consider that we are much indebted to Mr. Porter for giving us an appliance which is simple, inexpensive and efficacious. I may add, that, mixed with shot, so as to give weight to the appliance, these pads may be used to give pressure, when such is desirable, as over some forms of abscess, to prevent redistension from collection of pus in a sac which has been opened—*G. W. Callender in London Lancet.*

---

TREATMENT OF CROUPOUS PNEUMONIA.—Dr. Robinson, in a paper before the American Medical Association, says: In the absence of the author of the paper, Dr. A. B. Palmer, of Ann Arbor, Mich., it was read by Dr. N. S. Davis. The paper principally dealt with the effects of quinine in the treatment of acute pneumonia. After detailing the nature of the disease, and describing the various methods of treatment, Dr. Palmer submitted his particular method. When called to a patient within twelve or twenty-four hours after the chill, or at any time before any considerable exudation has occurred, he immediately gave from six to ten grains of quinine, together with from one-fourth to one-third of a grain of morphine, which almost invariably, in a short time, from half an hour to two hours, induced free perspiration, and a reduction of the temperature. Then he repeated the quinine in doses of from four to eight grains once in from two to three hours, and unless all pain and special uneasiness was relieved, he added another, but usually a smaller, dose of morphine in four or six hours, but by all means continuing the quinine in any of the last-mentioned cases until from thirty to fifty, and sometimes sixty, grains were given. Sometimes from twenty to twenty-five grains would be sufficient, given in these divided doses, or, if preferred, in doses somewhat smaller, but more frequently repeated. But as the larger quantity was harmless and might be needed, he preferred to give at least thirty, and oftener as much as forty grains in from twelve to twenty-four hours. The effect desired, and certainly as a rule produced, was a decided reduction of temperature, a marked diminution in the frequency of the pulse, a decided moisture of the skin or free sweating, a slower and more easy respiration, a relief from pain and the feeling of fullness in the chest, a diminution of the cough and of the tenacious and bloody character of the expectoration. In short, not only was there a checking of the fever, but of all the evidence, general and local, of the pulmonary engorgement and inflammation; the quantity of the medicine to be given depended much upon the completeness of the effects to be produced. The slight deafness and ringing in the ears, which might or might not result from these doses was a matter of very little consequence was almost always temporary, and should not influence the quantity given. A small quantity of quinine would produce these phenomena with some, while larger doses would fail to do so with others, and neither in pneumonia nor in ague were they the measure of the medicinal effect of the remedy, or an index of the quantity that would be required or borne. As a rule, all the treatment required after this was a gentle laxative, or if the tongue was much coated, a few grains of blue mass, followed in a few hours by a mild saline cathartic, and that in turn followed by some mild eliminative mixture.

[I have twice in my own case aborted pneumonia in the initial stage, characterized by pain in the chest and rigors, by going at once to bed, applying hot rocks to the extremities, a large mustard plaster around the entire chest, and taking a full dose of quinine and Dovers powder. The effect was speedy reaction, followed in a short time by diaphoreses and relief of pain. There was bloody expectoration in both cases, but no inflammatory action. A second smaller dose of the medicine was taken in both instances, and I kept my room about two days. —Ed. W.]

---

Collodion Flexile in Cases of Eczema.—Henry Lawson, M.D., Assistant Physician to, and Lecturer on Physiology in, St. Mary's Hospital, says: In my hands, two bad cases of eczema—E. genitale and E. capitis—collodion has shown itself so valuable a remedial agent that I lose no time in publishing the result, in order that others may try it, and see what the consequences are likely to be. I shall now describe one of the cases.

The first case was one of E. genitale. The patient, M. E——, was a woman aged about forty-seven years, married, and the mother of several children. She was a florid woman, of an active temperament, well nourished, of moderate habits of life, tolerably cleanly, and with a pulse strong and full and about 74 in the minute. She had lost her courses about two years ago; and, indeed, her general appearance was not such as led me to commiserate her very much. However, an examination of the patient showed that she had been suffering a good deal. The whole of the neighborhood of the perineum, of the parts about the vulva, and of the inner margin of both thighs, were covered with an eruption. And what was its nature? It is difficult to describe it. It had a reddish or reddish-purple aspect, which was, of course,caused by the injection of the parts with blood; and it could be seen that certain parts were slightly raised; while over the whole surface was a sort of semi-transparent glutinous liquid mass, with here and there some scaly particles of epidermis. It did not smell badly, though the entire amount of surface exposed must have been quite a square foot; but it was accompanied by great pain, heat, and secretion of liquid matter. Indeed, the patient declared that it made her life a perfect misery.

Well, I first tried tar water, and with some success, but not enough, for after a fortnight she was nearly as bad as on the first day I saw her, and she had been fourteen months suffering under this disease. So I resolved to try the collodion flexile. I placed her on the sofa, and proceeded to literally cover the diseased parts with collodion, and then I put a second layer over the first. I next directed her to put on this mate rial twice, or oftener, if needful, every day, and to come to me in a week and report progress. At the same time I forbade her to take tea, coffee or malt liquors, but to substitute cocoa or milk, and to take a little whisky if she desired it. Finally, I ordered her a compound colocynth pill, with podophylin, to be taken occasionally at night.

When, at the end of a week, this patient came to me, I was absolutely astounded at the progress she had made. There was not at all the same amount of secretion over the surface, and it seemed paler, while it had not extended in the least degree. She said she felt she was getting better, and that it was not nearly so painful as it had been. Of course I simply repeated the prescription, and when she came again in a fort night, all appearances of liquid on the surface had disappeared. The extent of the affected parts had diminished, so had the pain, which was now nearly *nil*. In fact, the remedy had acted most satisfactorily, and there was nothing to do but repeat it. This course was followed out by the patient for about two months, at the end of which she presented herself completely cured of the painful E. genitale.—*London Lancet.*

---

A New Instrument for the Administration of Compressed Air.—Dr. F. H. Davis, of Chicago, has devised a new instrument that is designed to be a cheap substitute for the more expensive ones, such as Waldenburg's, now in use. It consists of a small gasometer, fifteen inches in diameter by twenty-four inches in height, and should be made of galvanized iron. On the side of the outer tank two upright bands are soldered, embracing between them a slip of wood that reaches twelve inches above the tank, and to the extremity of which is hinged a wooden lever two feet long. A handle in the middle of the inner tank is attached to the middle of the wooden lever. But slight exertion on the part of the patient in raising or lowering this lever, produces all the pressure or suction necessary. The apparatus can be made by any tinsmith, and with three feet of three-quarter inch hose, costs from four to five dollars. The patient inspires the compressed air, or, *vice versa*, expires into rarefied air and inspires again the ordinary atmosphere. To obtain more marked result two gasometers are sometimes employed—one to inspire from, and one to expire into. As the fever is raised the patient expires into the one, the other being at the same time raised full of fresh air; he then inspires the fresh air as the lever is depressed, the foul air at the same time emptying itself from the first tank. One mouthpiece only need be used, and a light gauge may be attached to show the exact amount of pressure or suction. Fitted out in this way, the apparatus would cost from fifteen to twenty dollars. Dr. Davis has seen the advantage of this treatment in several classes of cases. It has been chiefly in those young persons of slight physique, whose chests were flat and narrow, and had deficient expansive power. While physical examination showed no tubercular deposits, there was a sensitive condition of the air-passages, causing frequent attacks of cold. In such persons the inhalation of compressed air from five to ten minutes, once or twice a day, produced very decided improvement. Under its use, in combination with some tonic, such as the malt extract, or cod liver oil, the patient's condition improved, his carriage became more erect, inspiration deepened, and the sensitiveness to catarrhal attacks lessened. Where there is incipient or primary tuberculosis, such as is shown by a loss of flesh and strength, with diminished appetite, shortness of breath on exercise, slight pains in the chest, usually a hacking cough, and expectoration of a mucous characacter, rather harsh bronchial respiration, and more or less dullness on percussion, it is thought that the question of improvement under compressed air will depend a good deal upon the hereditary taint. When the phthisis is *inflammatory* in its origin, the best of results may be anticipated, unless the stage of softening has commenced, and then this treatment should not be attempted for fear of hemorrhage. In emphysema and valvular diseases of the heart, the author has had no experience.—*Chicago Med. Jour. and Examiner*, *Oct.* 1877.

---

THE TREATMENT OF PULMONARY CAVITIES.—The following important suggestions are from an article in the London *Medical Times and Gazette*, by Dr. R. D. Powell, of the Brompton Hospital for Consumptives: In the treatment of secreting cavities, the objects we have in view are (1) to lessen secretion, (2) to promote evacuation of what secretion is formed, and (3) to disinfect such cavities. Counter-irritation, of little use, I believe, whilst cavities are still forming or extending, is of great service in these cases. When we remember that in chronic ex–cavation of the lung we almost invariably get an intimate union of the two pleural surfaces, and an anastomosis of their vessels, we may see why the application of a blister externally may affect such cavities. As a matter of fact, they do influence them most decidedly. Strong iodine applications (two drachms to the ounce), or fly blisters, or perhaps a blister kept open for a few days by the use of savin ointment dressing, are the forms of counter-irritation suitable to different cases. Under their use the cough and expectoration frequently diminish. Acids and astringent iron tonics and oil are needed. Sedative cough mixtures are directly contra–indicated in these cases, except for the purpose of giving rest at bedtime. It is in these cases the inhalations are most useful; for, firstly, there being no actively spreading disease present they can do no harm; secondly, we can, by their use, render less noxious the pus that bathes the surface of the cavity, and which is apt to become inhaled during the effort of expectoration into distant parts of the lung: thirdly, inhalations help expectoration; fourthly, there can be little doubt that appropriate inhalation sometimes have a healing or an alterative effect upon the internal surface of the cavity.

The best substances for inhalation are —iodine (vapor iodi, B. P.,) only to be used occasionally and for a few days together; carbolic acid (glycerine of carbolic acid, one drachm to two drachm, to half a pint of hot water;) or tar water (liq. carbonis detergens, one drachm, to half a pint of water:) useful disinfecting and, except iodine, somewhat sedative inhalations, that may be employed two or three times a day. They may be taken very well from a deep jug, or a Nelson's inhaler, with the sponge removed. Friar's balsam, tincture of larch, turpentine, etc., may be similarly employed from time to time. Perchloride of iron or other astringents may sometimes be used with Siegle's spray apparatus; but I have myself failed to find atomized astringents useful in these cases, and doubt if they penetrate so far as vapors inhaled in the ordinary way. Salt air, and perhaps especially sea-shore air, containing more or less salt spray, is nsually beneficial to these patients; some liberal diet is, of course, necessary.

Medicinaily these cases my be combated by quinine, internally, or in some cases full doses of perchloride of iron, or sedative inhalations containing tincture of benzoin and opium; hyoscyamus and chloric either, carbolic acid and opium, etc., are useful. Ipecacuanha wine, administered as spray, with Siegle's inhaler, is worth a trial, but patients suffering from this condition of cavities are often too prostrate to bear the fatigue of inhaling. If the more active general symptoms should lessen, but a blood-stained and copious expectoration still leads us to infer that the walls of the cavity are hyperæmic, I am convinced, from observation, that the best treatment is to apply a blister over the region of the excavation, and to keep it freely discharging for several days, by means of savin ointment dressing. I have seen active symptoms completely subside under this treatment—which is, however, somewhat severe and painful—and the cavity subsequently contract, the expectoration, from being abundant and sanguineous, becoming scanty, viscid, and apparently consisting of bronchial mucus only.—*Medical & Surgical Reporter.*

---

Colocynth for Abdominal Pain.—Dr. James I. Tucker writes to the Chicago Medical Journal and Examiner: "I state without fear of successful controversion that colocynth will allay the pain caused by excessive peristaltic action better than any drug in use, not excepting opium, provided it be used in the proper dose. I refer to simple but nevertheless distressing idiopathic pain, so to speak: pain due to excessive stimulation of the nerves engaged in keeping up the harmonious rythm of the vermicular movement of the bowels. In such cases I employ not the solid extract, but the *tincture;* and I use the tincture in such small quantities that I expect to meet a large amount of incredulity growing out of *a priori* conclusions. But why, pray, if ipecac in minute doses can allay nausea and vomiting, may not colocynth in small doses allay the very griping which in large doses it is capable of producing? I use only just so much of the tincture as to render the excipient—generally water—slightly bitter. In teaspoonful doses, repeated *pro re nata,* I have seen the most speedy relief from very violent griping. Now, since the therapeutics is the ultimate aim of classical or humanitarian medicine, I hope much more attention wili be paid hereafter to the hitherto unutilized virtues of drugs which have been supposed to have but a very limited applicability. It will be found that our methods of ascertaining the therapeutical possibilities of drugs are lamentably meagre, and without honest original research we bow too willingly to the shrine of supposititious authority. The truly medicinal properties of many of the drugs in common use lie latent, dormant, and neglected, ready at any time to grow and bud and blossom, like the germinal principle which was at last discovered in the wheat grains found in the Egyptian catacombs. It is the duty of every practitioner to contribute the results of his experience to the common store of knowledge; not, indeed, to tell us what misery he can occasion by doses of this or that, but how far this or that has contributed, by a careful artistic application, to alleviate the sufferings of mankind. The basis of observation has been hitherto very inadequate; but the time is coming—nay, is already here—when the action of drugs may be ascertained with mathematical accuracy. I mean by the neurological method of therapeutics. To this fact, and to the other virtues of the bitter cucumber, which are an illustration of this fact, I now endeavor to call the attention of the medical profession. Therapeutics resting on a neurological basis is to be the therapeutics of the future.—*Louisville Medical News.*

How the Chinese Make Eunuchs.—The following curious description is given by a writer in the *Lancet*:

The operation is performed at an establishment maintained for the purpose, immediately outside one of the palace gates. The operators are known as "knifers," and they contrive to keep the trade in their own families. For each castration, and the subsequent care of the ease, they receive the equivalent of about £1 16s. sterling. When about to be operated on, the patient is placed in a semi-supine position, on a broad bench. One man, squatting behind him, grasps his waist, and one man is told off to each of his legs. Bandages are fastened tightly round the hypogastric and inguinal regions, the penis and scrotum are three times bathed with a hot decoction of pepper.pods, and the patient is finally, *if an adult*, solemnly asked whether he will ever repent. If he appears doubtful, he is unbound and dismissed, but if his courage has held out, as it usually has, all the parts are swiftly swept away by one stroke of a small sickle-shaped knife. A pewter plug is inserted into the urethra, the wound is covered with paper soaked in cold water, and is firmly bound up. The patient, supported by two men, is kept walking round the room for two or three hours, after which he is permitted to lie down. For three days he gets nothing to drink, nor is he allowed to pass urine At the end of this period the dressings are removed, and the plug is taken out. The parts generally heal in about one hundred days, when the patient is inspected by an old and experienced eunuch, in order to make sure that the mutilation is complete. About two per cent. of all cases prove fatal, some by hemorrhage and some by extravasation. For a long time after the operation there is nocturnal incontinence of urine.—*Medical & Surgical Reporter.*

Mental Emotion as a Cause of Impotence.—Now, there are no organs in the body so readily influenced by mental emotions as the genital organs, and mental emotion is one of the commonest causes of impotence in existence. Impotence of this nature may occur in married or single men in comparative health, as well as in those who have masturbated to excess. In the latter it is more apt to take place, but you will find very many cases where there is no other assignable cause but mental emotion. Troubles in business, anxiety about the health, family jars, a fit of indigestion, a disagreeable remark from one of the interested parties, a leucorrhœal discharge with a fetid odor—in fact, any uncomfortable feeling whatever which takes the mind away from the act, will bring on impotence; and, when one failure occurs, no matter how trivial the cause, others are sure to follow. Fear is an essential element in many cases—fear that the act cannot be accomplished; it is this fear which produces impotence in many who have masturbated. They read in advertisements and elsewhere of their loss of manhood, and the very first attempt at intercourse fails, even when there is really no loss of power. Temporary impotence has been produced in a healthy man by a friend's recital of his own surprising failure. The thought of the accident which befel the friend occurred at the wrong time, and he too failed.

I might go on with innumerable instances of such cases of impotence, but our time will not admit of it; neither do I think it necessary. But I do wish you to be impressed with the idea that emotions cause impotence in most patients, and that such impotence is always amenable to treatment, and can, with rare exceptions, be recovered from. —*Prof. Howe in Med. Record.*

Veratrum Viride Poisoning.—Dr. Elrich. This was occasioned by the reading of Dr. Lynch's paper before the Medico-Chirurgical Faculty, on the subject of veratrum. Years ago I had used this drug, and had such unpleasant results follow that I abandoned it, but Dr. Lynch induced me to give it another trial. A child, sixteen months old, had membranous croup, so far as I could determine, although there was no false membrane discharged, nor patches on the fauces. Two drops were ordered every two hours. After taking a few doses it vomited, and the breathing was relieved. One morning the mother gave a teasponful in place of a cough mixture; it commenced vomiting soon after, its extremities became cold, a cold clammy perspiration appeared, and it seemed to be in collapse. As Dr. Lynch recommended opium as the antidote for any unfavorable symptoms, I injected three drops of deodorized tincture of opium, and in a short time the alarming condition disappeared.

Dr. Wintermitz. It will invariably act in that manner unless combined with an opiate. If an opiate had been given with it, no ill effect would have been seen.

Dr. Rohe. I hope Dr. Wintermitz does not recommend teaspoonful doses, even when combined with opium.

Dr. Seldner. In spite of its combination with opium, as soon as the pulse is reduced, it will produce emesis of such obstinate and depressing character, that I have abandoned its use.

[For more than twenty-five years we have used *Norwood's tincture veratrum.* Once, by mistake of a nurse, twenty-drop doses were given every two hours, until two drachms were taken, with no other effect than frequent vomiting, and a degree of prostration not so great as would have resulted from emetic doses of lobelia. While this fact shows that viratrum is not so dangerous and poisonous as has been supposed, yet it is not necessary or advisable to give large doses, when smaller ones will accomplish the object for which it is usually prescribed, to-wit: the reduction of the pulse. We never fail to accomplish this with two to four-drop doses, given every two hours, to an adult. Given thus, it rarely nauseates the stomach. It is true, as above stated, that opium counteracts the tendency of the remedy to produce nausea; and yet it does not prevent its sedative action upon the circulation.—Ed. W.]

---

Chloral Drinker.—"In June, 1875, I thought chloral hydrate might be something nice, I looked over all the Druggists' Circulars at hand, and concluded that it was harmless in thirty grain doses. As we had none in the store, I went to another drug-store and purchased one ounce. I took thirty grains of it in a tumblerful of water, which had a pleasing effect. I then took thirty grains more, which seemed to take away my memory. I followed it up for two days, every little while taking thirty grains largely diluted with water. During those two days I swallowed three-fourths of an ounce of chloral hydrate. At last I could not hold anything in my hands, which were partially paralyzed. I had to be assisted home, and went to bed and slept most of the time for one day and two nights. I then went about my business, but of all the sufferings I ever endured I think this was the worst. I was not free from pain a moment for thirty days. The pain was greatest in my knees and legs. I would go to bed at night, get in an easy position, and lie perfectly still, and not stir in the least, and finally would go to sleep. The first thing on awaking were those dreadful pains. I took nux vomica and phosphorus pills but they did me no good; the disease had to wear itself out. The sufferings resembled those of the opium-eater when deprived of the drug. With one or two exceptions, I can not recall any thing that transpired during those two days. I think that I had a narrow escape from death. I shall not take any more chloral hydrate."—*Drug. Circ.*

---

Cold and Sweating Feet.—Dr. Rumbold in Virginia Medical Monthly, says: If there is fœtor from the feet, salicylic acid and bromide of potassium: aa grs. v ad. oz.j of "vaseline," will, in a few bathings and annointings, correct this condition.

Plunging the warm feet in cool water immediately on getting out of bed in the morning, has, frequently, a good effect.

Boots that are thin or tight, and shoes that are low in the ankles, should be avoided in cold or damp weather. Heavy, loose-fitting boots, with double uppers and soles—the latter made wide—are the proper coverings for the feet, in cold or damp weather.

India-rubber overshoes should be worn in wet or damp weather only, and they should be removed from the feet as soon as the wearer enters the house.

Slippers should not be worn by either sex during cold or even cool weather. One of the ways in which a cold is *mysteriously* (?) contracted, is to exchange a pair of warm boots for a pair of low slippers. Those who do this have forgotten that their feet and ankles had been protected all day, and that they have not only uncovered them, but placed them in the coldest stratum of air in the room. If they had taken the precaution to draw on over the stockings which they usually wear a pair of heavy woolen socks, the chances for taking cold from wearing the slippers would have been greatly decreased.

---

Diagnosis of Hip-diseases in Children.—In examining a child suspected to have hip-disease, be careful to place him on something firm and flat—a table covered with a blanket, a leather couch, or the floor. If you use a soft bed, he will sink into it, and you will perhaps overlook even a considerable deformity. Do not be content with any thing short of a thorough examination. Do not pretend to say whether a child whom you have examined, with his trowsers on, has or has not hip-disease. Let him be undressed, so that you can move his limbs without being hindered by his clothes. Girls past early child-hood may be fully examined, if you use a shawl or a loose sheet to cover them. 1. You must look for abnormal posture of the limb or of the pelvis; 2. For stiffness at the joint; 3. Observe whether the glutei, or the muscles of the thigh, are wasted, or whether any, especially the abductors, are rigid; 4. Or whether there is any swelling about the joint or in the thigh or the iliac fossa; 5. Notice the relation of the trochanter to the side of the pelvis as compared with that of the opposite side; 6. Look to the length of the limb as compared with that of its fellow; 7. See how the patient walks, if he be able to do so; 8. If he have pain, learn its situation and its character.—*Howard Marsh, in British Medical Journal.*

---

Masturbation.—There are certain gymnastic exercises which are provocative of masturbation, and which should be prohibited until manhood is reached. These exercises, too, are common in all our school gymnasiums, as well as public gymnasiums. My attention was first directed to this important fact, a few years ago, by the history which a confirmed masturbator gave me of his first experiences. When a lad of eight he commenced exercising in the school gymnasium. One day, while using the swinging pole, sustaining the whole weight of his body by his hands, he felt a peculiar sensation in his genital organs, which became so intense that he was compelled to sit down. Every time he resumed the exercise the same pleasurable sensations returned. They led him to a closer examination of his genital organs, and subsequently to new methods of increasing the excitement, until he became a confirmed masturbator. Since then, I have had a somewhat similar history from five other persons, one a female, confirming the opinion which I I now hold, viz.: that all exercises, such as climbing and sliding down poles, swinging from rings or ropes—the body being supported by the hands alone—should be dispensed with until the age of twenty or thereabouts; then there is less danger of determination of blood to the genitals as a consequence of these exercises.—*Prof. Howe in Med. Rec.*

---

Schiff on the Functions of the Spleen.—The spleen appears to incease in volume from the fourth to the seventh hour of gastric digestion. During the digestion, or rather during gastric absorption, the spleen prepares the ferment, which, entering with the blood into the tissue of the pancreas, forms in this latter a special substance into pancreato-pepsine or trypsine, a material suited to digest albuminoid bodies. After extirpation of the spleen, the pancreatic juice loses its digestive power on the albuminoid bodies, though it preserves its other digestive properties. The duodenal digestion of albuminoid substances is no longer marked by its energy and rapidity; it becomes weak just as in other parts of the small intestine After removing the spleen,the matter destined to form the pancreato-pepsine accumulates in large quantity in the pancreas,and can still be transformed into pancreato-pepsine by those chemical influences which after death attend the commencement of putrefaction. After the nerves of the spleen have been destroyed, the organ remains flaccid; it no longer becomes tumid, but atrophies, just like the erectile tissues when the vascular nerves have been paralyzed.—*La France Medicale*, 79, 1877.

Abortive Power of Alcohol in Diphtheria.—I was called on a Sunday evening by my friend, Dr. Burge, to see a lady whose child had died the previous morning, of diphtheria. She had held the child most of the time of its illness, and kissed it repeatedly and frantically on the lips, as putrescent matter flowed from them in its last moments. Friday and Saturday she had had an exudation of moderate extent in the fauces, but now she had, in addition, a diffused intumescence and induration of the neck. Seeing that the patient had inhaled the child's breath for six days, had had patches on both tonsils for two, and had applied her mouth to the poison many times, I mentioned to the doctor that we had all the conditions present foreshadowing a malignant case, and that regarding alcohol as a true antidote, I would advise its administration in full doses, the same as when the disease was at its height. Besides the quinine previously prescribed, the patient took a tablespoonful of whisky every hour with this result: she attended the child's funeral on Tuesday, and in a day or two regained her usual health. In this case, the abortive power of alcohol was fully manifested—a power it rarely fails to assert.—*Dr. Chapman in Society County Kings.*

The American Pharmaceutical Association held its Twenty-fifth Convention at Toronto, Sept. 4th to 7th. Brooklyn was represented by Messrs. Cutts, Heydenreich, Sayre, Menninger and Dr. Squibb. The action taken by this convention concerning pharmacopœial revision was diametrically opposite to that taken by the American Medical Association at Chicago. Not only did it ordain the appointment of a strong committee who are to prepare the text of a modernized pharmacopœia, but it tendered to Dr. Squibb a vote of thanks "for his earnest efforts during the past two or three years to inaugurate an improvement in the plan of revision." This resolution, introduced by Mr. Sheppard, of Boston, was affirmed unanimously. In this connection, *The Druggists' Circular* says: "The project devised by Dr. Squibb was treated by the A. M. A. with undeserved harshness, both towards the project and its projector—in fact, in a manner that was but little short of contemptible."...... The committee above referred to is composed of Charles Rice, Chairman; F. Hoffman, Bedford Maisch, Remington, Bullock, Markoe, Sheppard, Hancock, Ebert, Diehl, Wayne, Crawford, Mohr, Painter, Saunders.—*Society Co. Kings.*

When Not to Give Iron.—In the current number of the Practitioner Dr. Milner Fothergill has contributed a few practical remarks on the contra-indications for giving this drug. As long, he says, as there is rapidity of pulse combined with rise of temperature, so long must iron be withheld in the treatment of acute disease. As long, moreover, as the tongue is thickly coated, or red and irritable, it is well to withhold chalybeates altogether. This is particularly true of phthisis. No matter what the other indications are, it is useless and sometimes worse than useless to give it without the tongue be clean without irritability.

It may be laid down as a general rule that this toleration of iron diminishes as the age increases. Young children take iron well, and it is often well borne by them in conditions which in the adult distinctly forbid its use.

There is one condition where iron is absolutely forbidden, and that is in the condition known as biliousness. As long as there is a foul tongue, a bad taste in the mouth, and fullness of the liver, with disturbances of the alimentary canal, iron is not only of no service, but positively does harm.—*Louisville Medical News.*

---

CYANIDE OF ZINC IN RHEUMATISMAL NEURALGIA.—Dr. Lutton, of Rheims (*Bull. Gen. de Therap.*), again calls attention to the value of the cyanides in the treatment of rheumatism. He gives notes of two cases, one of sciatica, followed by trifacial neuralgia and delirium, where the remedy was administered according to the following formula:

℞ Zinci cyanid.........3 grains.

Aq. destillatæ.........6½ drachms.

Mix. Mucilag. acaciæ......4 drachms.

Sig.—Tablespoonful every hour. Shake well before using.

The effect produced was surprising. The patient suffered less the first day after commencing treatment, the accompanying fever abated, the pain became tolerable, sleep and appetite returned. Within three days the disease was cured, and did not return. A second case of trifacial neuralgia, accompanied by acute articular rheumatism, fever, cerebral trouble, was cured rapidly by the same means. Dr. Lutton gives notes of both of these cases. In the remarks which follow, he takes occasion to complain of the unmerited neglect with which this remedy has been treated by the profession, and complains almost bitterly of the popularity of propylamine and salicylic acid. —*Philadelphia Medical Times.*

---

PATENT NOSTRUMS.—*R. R. R.* consists of a reddish yellow liquid which smells of ammonia and camphor. Contains 14 parts of soap, 40 parts of ten per cent. ammonia, 640 parts of alcoholic extract of cayenne or Spanish pepper, 4 parts of camphor, and 2 parts of oil of rosemary.

*Mrs. Winslow's Soothing Syrup* consists, says Hager, of 8 parts of white simple syrup mixed with 1 part of a tincture made by extracting 10 parts of freshly crushed fennel seed and 1 part of oil of fennel white 60 per cent. spirits.

*Sozodont.*—This reddish liquid consists of a solution of 5 grammes of oil soap in 6 grammes of glycerine, 30 grammes of spirit, 20 grammes of water, perfumed with a few drops of oil of peppermint, oil of cloves, oil of cinnamon, and oil of anise colored with cochineal. The powder is a mixture of carbonate of lime, magnesia, and orris root.

*Worm Lozenges* contain 1 part of calomel, 6 parts of santonin, and 290 parts of sugar.

*World's Hair Restorer* contains, says Wittstein, 5.6 grammes of sulphur, 8 grammes of sugar of lead, 100 grammes of glycerine, and 200 grammes of aromatic perfumed water.—*New Remedies.*

---

WHEN SHALL THE LYING-IN WOMAN GET UP?—In a discussion upon the above subject before the Society of the County of Kings, Dr. B. M. Briggs remarked:—

Flexions and other uterine complications will follow too long rest, just as truly as they follow a premature getting up. His practice is to advise a gradual assumption of the upright posture, beginning generally on the fifth day. The patient is not to lift anything heavy, or do hard work for a fortnight. . . The too common practice is to make unnecessary changes in the diet; he orders a diet almost similar to that taken before delivery.—*Med. Society County Kings.*

